# Chemokine receptors as important regulators of pathogenesis during arboviral encephalitis

**DOI:** 10.3389/fncel.2014.00264

**Published:** 2014-09-30

**Authors:** Daniela Michlmayr, Jean K. Lim

**Affiliations:** Department of Microbiology, Icahn School of Medicine at Mount Sinai, New York, NYUSA

**Keywords:** chemokine receptors, flaviviruses, alphaviruses, leukocyte infiltration, antagonists

## Abstract

The central nervous system (CNS) is a highly complex network comprising long-lived neurons and glial cells. Accordingly, numerous mechanisms have evolved to tightly regulate the initiation of inflammatory responses within the brain. Under neuroinflammatory conditions, as in the case of viral encephalitides, the infiltration of leukocytes is often required for efficient viral clearance and recovery. The orchestration of leukocyte migration into the inflamed CNS is largely coordinated by a large family of chemotactic cytokines and their receptors. In this review, we will summarize our current understanding of how chemokines promote protection or pathogenesis during arbovirus induced encephalitis, focusing on neurotropic flaviviruses and alphaviruses. Furthermore, we will highlight the latest developments in chemokine and chemokine receptor based drugs that could have potential as therapeutics and have been shown to play a pivotal role in shaping the outcome of disease.

## INTRODUCTION

Arboviruses (arthropod-borne viruses) are a significant cause of human morbidity and mortality and have worldwide distribution. In recent years, these viruses have become an increasing public health concern due to climate change, increased globalization, and other environmental factors, that have caused their unexpected geographic expansion and increased the frequency of outbreaks (Gubler, 1996). The World Health Organization has estimated that arboviral infections constitute ∼30% of all emerging infectious diseases in the past decade (Jones et al., 2008). The most recent and well-documented examples include the introduction and spread of WNV in North America in 1999 and the continuing emergence of CHIKV in the regions of the Indian Ocean in 2005/2006 (Hayes et al., 2005; Bonn, 2006).

Neurotropic arboviruses have the capacity to enter the CNS and cause inflammation and severe neurologic sequelae in humans. Many of these viruses are members of the *Flavivirus* (*Flaviviridae* family) and *Alphavirus* (*Togaviridae* family) genera. The main perpetrators of arboviral infections in humans include JEV, with 30,000–50,000 cases reported annually, WNV, and TBEV (Campbell et al., 2011). Mosquito-borne alphaviruses are also important causes of encephalomyelitis and include WEEV, EEEV, and VEEV. SFV and SINV are neurotropic viruses that do not usually cause encephalitis in humans, but are studied frequently in mice as model systems for alphavirus-induced encephalomyelitis.

Acute viral encephalitis is a life-threatening condition that is characterized by the presence of leukocytes within the brain parenchyma. Viral replication within the CNS can lead to neuronal damage and results in apoptosis and necrosis of these cells. As part of innate and adaptive immune responses to viral replication, a large number of leukocytes infiltrate the CNS, and the cell types and composition of the inflammatory response can vary greatly between individuals and between pathogens. The large influx of leukocytes into the normally immune-sheltered CNS is required for recovery and clearance of virus but is often associated with neuropathology (Hosking and Lane, 2010; Ransohoff and Engelhardt, 2012).

Chemokines play a pivotal role in the attraction of leukocytes into the CNS, and it is imperative to understand their cell-type specific role in pathogenesis in order to develop novel immunotherapeutics and predict the impact of chemokine receptor antagonism in humans. Chemokines and their receptors comprise a large superfamily of proteins that can be categorized into four subfamilies based on the position of the first two cysteines within the first amino terminal cysteine motif: CC, CXC, XC, and CX_3_C (Zlotnik and Yoshie, 2000). All chemokine receptors are G-protein coupled receptors, containing a seven-transmembrane domain that interacts with the appropriate chemokine upon binding. Chemokines and chemokine receptors have been shown to have pivotal roles in organizing and coordinating complex immune system functions (Zlotnik and Yoshie, 2012). Many studies have been conducted in the past to elucidate the role of chemokines during viral encephalitis. In this review, we will summarize the role of chemokines and their receptors specifically during arbovirus induced encephalitis. In particular, we will focus on WNV, JEV, TBEV, SFV, and SINV, as these pathogens are the most studied in the context of chemokine-mediated leukocyte infiltration into the virally infected CNS in both mouse models and humans. Furthermore, we will also highlight chemokine receptor based drugs that are either approved or in development for human use, as well as chemokine specific antibodies, and their anticipated effect in the context of human arboviral encephalitis.

## IMMUNE RESPONSES IN THE CNS DURING ARBOVIRAL ENCEPHALITIS

From an immunological point of view, the CNS is a unique compartment due to the following features: lack of antigen presenting cells, low expression of MHC I and MHC-II, lack of lymphatic vessels within the brain, absence of resident DC, BBB, and BCSFB that restrict entry of cells and substances into the CNS (Ransohoff et al., 2003). If the BBB is compromised due to infection or inflammation, immune cells are able to infiltrate the brain (Rivest, 2009). Despite the mostly effective host responses during early stages of viral infection, controlling viral spread within the CNS requires the influx of peripheral leukocytes that can often cause profound damage to neurons and glial cells. Therefore, immune responses within the host must be balanced as to prevent damage to delicate and mostly non-renewable neurons.

Neurotropic arboviruses replicate in the periphery prior to entry and replication in the tissue of the CNS. Within peripheral organs or lymphoid tissues, the elicited immune response is often sufficient to prevent viral entry into the CNS. In fact, most infections with flaviviruses are asymptomatic/subclinical, with no evidence of neuroinvasion (Mostashari et al., 2001). However, if the virus enters the CNS, the infected target cells as well as bystander cells produce numerous chemokines and cytokines, which in turn initiate neuroinflammation (Neumann, 2001). Based on several RNA based assays, some of the chemokines produced within the CNS during arboviral encephalitis are CCL1–5, CCL7, CCL8, CCL12, CXCL1, CXCL2, and CXCL9–13 (Gupta and Rao, 2011; Yang et al., 2011; Metcalf et al., 2013; Palus et al., 2013; Michlmayr et al., 2014). In particular CCL2–CCL5 and CXCL10 are consistently and highly induced during JEV, WNV, TBEV, SFV, and SINV infection. In addition to infected neurons, activated astrocytes and microglia are also a major source of chemokines within the inflamed brain. Our study and a study by Shirato et al. has revealed that the extent of chemokine expression during WNV infection is dependent on viral strain and severity of the disease (Shirato et al., 2004; Michlmayr et al., 2014). Another study with TBEV has shown that mice highly susceptible to TBEV infection display a higher fold induction of chemokine transcripts and low levels of neutralizing antibodies compared to mice with low susceptibility to TBEV infection. Thus, the extent of the host response may be positively correlated with pathogenesis of TBEV infection in mice (Palus et al., 2013).

The leukocytic infiltrate and its role within the CNS during viral encephalitis is dependent on numerous factors, including the infected cell type, the route of infection, and the strain and inoculum of virus. Evaluation of human and mouse CNS tissue indicates that neutrophils, CD4^+^ and CD8^+^ T-cells, and monocytes/macrophages are typically present during viral infection (Parsons and Webb, 1982; Johnson et al., 1986; Williamson et al., 1991; Holub et al., 2002; Samuel and Diamond, 2006). Currently, the role of neutrophils during neurotropic viral infections, both in the periphery and within the CNS, is unclear. Antibody depletion of neutrophils results in variable outcomes, depending on the virus used and timing of administration; the receptors used to enter the CNS have not been studied. T-cells, in particular CD8^+^ T-cells, are a critical component of the cellular infiltrate into the brain (Shrestha et al., 2005; Sitati and Diamond, 2006), and blockade of T-cell entry into virally infected brains often results in changes in CNS viral loads and survival in mice. Whether T-cells are protective or pathogenic during viral encephalitis depends on the virus. Whereas T-cells play a predominantly protective role during WNV infection by mediating viral clearance in a perforin and granzyme-dependent manner (Shrestha et al., 2005; Sitati and Diamond, 2006), T-cells play a pathogenic role during TBEV infection, since CD8^-/-^ mice and SCID mice showed increased survival (Růžek et al., 2009). For monocytes/macrophages, it is clear that these cells do function in regulating pathogenesis, but this appears to be highly dependent on the strain of virus used, as well as the model of infection. Thus, it appears that the biological roles of specific cell subsets and the complex signals involved in their migration and function are highly dependent on the context of infection, as well as the virus itself. In this review, we will summarize the current understanding of the chemokine receptors CCR2, CCR5, CXCR2, CXCR3, and CXCR4 during flavivirus and alphavirus infection, focusing on the effect of therapeutic blockade of these receptors using small molecule receptor antagonists or chemokine neutralizing antibodies.

## THE ROLE OF CHEMOKINE RECEPTORS IN THE PATHOGENESIS OF ARBOVIRAL ENCEPHALITIS

### CCR2

**FIGURE 1:**
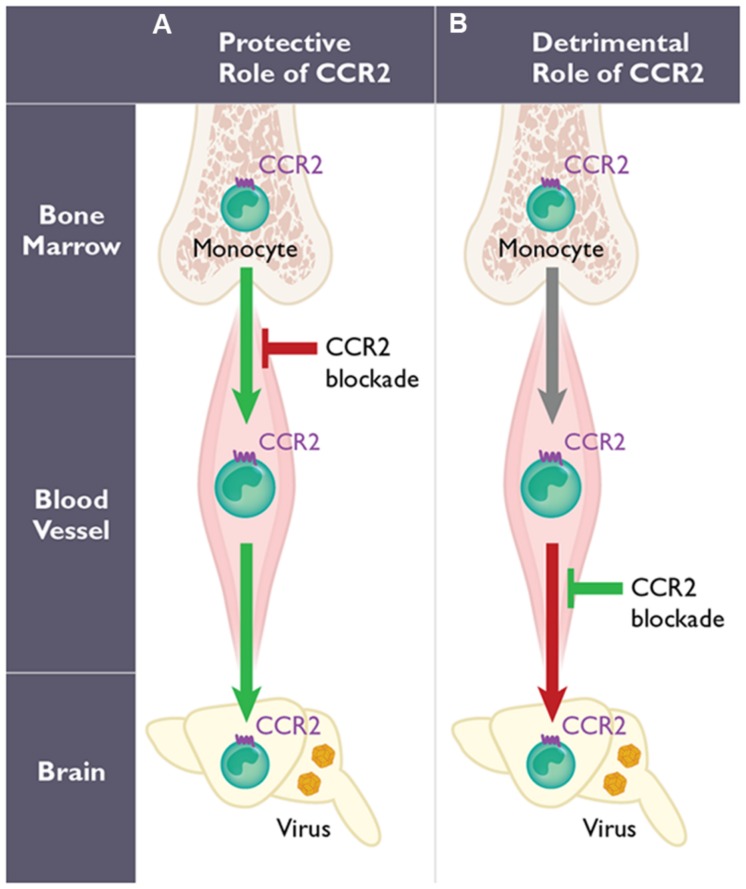
**The dual role of CCR2-expressing monocytes during West Nile virus (WNV) infection**. The function of CCR2 on monocytes may involve two distinct steps: egress from the bone marrow induced early following WNV infection, and their migration into the WNV-infected central nervous system (CNS). However, the role of CCR2-expressing monocytes is highly dependent on the mode of infection utilized in mouse models. With peripheral infection **(A)**, WNV induces a monocytosis within several days post infection. Genetic deficiency or pharmacological blockade of CCR2 in mice (red arrow) results in monocytopenia and leads to decreased monocyte infiltration into the CNS, enhanced viral titers in the brain and increased mortality. Therefore, CCR2-mediated monocytosis and the subsequent migration of monocytes into the inflamed CNS are protective (green arrows). In contrast using an intranasal infection model of WNV infection **(B)**, the migration of CCR2-expressing monocytes into the CNS has also been shown to promote pathogenesis (red arrow) in mice. Thus, blocking CCR2 using anti-CCL2 antibodies in this model prolongs survival (green) in mice.

CCR2 is often considered to be a receptor associated with monocyte trafficking, which is consistent with its high and uniform expression on Ly6c^hi^ “inflammatory” monocytes (Mack et al., 2001; Geissmann et al., 2003). This receptor is also found on subsets of activated T-cells, DC, and NK cells. In humans, a functionally equivalent “inflammatory” monocyte subset, identified as CD14^+^CD16^-^ also uniformly expresses CCR2 (Auffray et al., 2009). The primary and specific ligand for CCR2 is CCL2, but this receptor can also bind to ligands CCL7 and CCL12/CCL13 (Zlotnik and Yoshie, 2000). CCR2 has been postulated to be critical for the migration of monocytes into tissues during various inflammatory conditions (**Figure 1**). Recently, a novel role for CCR2 in regulating monocyte egress from the bone marrow under homeostatic and inflammatory conditions has been identified (Serbina and Pamer, 2006; Tsou et al., 2007). Thus genetic deficiency of CCR2 results in severe monocytopenia that could account for the partial or entire loss of these cells in inflamed tissues. The mechanism by which CCR2 modulates monocyte egress from the bone marrow into blood involves stromal cell sensing of TLR ligands, including those involved in viral sensing within the bone marrow. This results in the production of CCL2 within hours after infection, thus providing a mechanism by which systemic infections can trigger monocyte release (Shi et al., 2011). Thus, CCR2 and its ligands CCL2 and CCL7 are critical for modulating circulating monocyte numbers, and the additional role of this receptor in mediating migration from the blood into inflamed organs like the CNS, may be context-dependent (Shi et al., 2011).

Monocytes appear to have a critical role during WNV infection, although whether these cells function to protect or promote pathogenesis is unclear. Several studies have evaluated the role of monocytes using clodronate-loaded liposomes. In the first study, mice were infected intraperitoneally using a non-neuroinvasive strain of WNV, and monocyte depletion resulted in increased viremia, enhanced viral entry into the CNS, and increased mortality (Ben-Nathan et al., 1996). Another study, using subcutaneous inoculation of a neurotropic strain of WNV, showed a significant increase in peripheral and CNS viral loads and increased mortality following clodronate treatment (Purtha et al., 2008). Both studies imply that monocytes play a protective role in the context of WNV encephalitis (**Figure 1**). In a third study that utilized a lethal intranasal model and a non-neurotropic strain of WNV, depletion of peripheral monocytes, after treatment with clodronate, resulted in a reduction of CD11b^+^ cells in the WNV-infected brain during WNV encephalitis (Getts et al., 2008). The authors blocked Ly6c^hi^ monocyte migration into the CNS using an anti-CCL2 antibody and found a prolonged survival time compared to isotype-treated mice (**Table 1**), suggesting that inflammatory monocytes may function pathogenically during WNV encephalitis in this model (**Figure 1**).

**Table 1:** Inhibitors of chemokines/receptors and their role in arboviral encephalitis pathogenesis.

Blockade	Compound or antibody	Pathogen model	Role of antagonist	Reference
**Receptor antagonists**
**CCR2**	RS504393	SFV	No effect	Michlmayr et al. (2014)
**CCR5**	DAPTA	SFV	No effect	Michlmayr et al. (2014)
**CXCR3**	Compound 21	SFV	No effect	Michlmayr et al. (2014)
**CXCR3 & CCR2**	Compound 21 and RS504393	SFV	Beneficial	Michlmayr et al. (2014)
**CXCR4**	AMD3100	WNV	Beneficial	McCandless et al. (2008)
**Antibodies**
**CXCL10**	1F11 or 1B9	WNV	Pathogenic	Klein et al. (2005)
**CCL2**	2H5	WNV	Beneficial	Getts et al. (2008)

*The role of chemokine/receptor blockade can be either beneficial, pathogenic or have no effect on disease outcome in murine models of encephalitis.*

Using *Ccr2*-deficient mice, which are monocytopenic, we observed higher mortality and enhanced viral titers in the CNS during WNV infection compared to wild type mice, supporting a protective role of CCR2 (Lim et al., 2011). Detailed analysis of monocytosis revealed that WNV induces an approximate fivefold increase in Ly6c^hi^ monocytes in the blood within the first 5 days post infection. This response was found to be entirely dependent on CCR2, since *Ccr2^-/-^* mice showed no increase in monocyte levels in the blood throughout the course of infection. Within the CNS, a specific loss of Ly6c^hi^ monocytes was observed, while other infiltrating leukocyte subsets (CD4^+^ and CD8^+^ T-cells, neutrophils, and NK cells) remained unchanged compared to wild type controls, correlating the specific loss of inflammatory monocyte accumulation in the CNS with increased mortality. Adoptive transfer of monocytes into WNV-infected *Ccr2^-/-^* mice showed that both CCR2-expressing and CCR2-deficient monocytes were capable of entering the CNS, suggesting monocyte migration into the CNS is CCR2-independent.

Because of the significant role of CCR2 in monocytosis during WNV infection, the induction of its ligands, CCL2, CCL7, and CCL12/CCL13 is implicit. Indeed, CCL2 is detected in the plasma of WNV-infected blood donors during the acute phase of infection, and numerous studies have also shown strong induction of CCL2 *in vitro* (Tobler et al., 2008; Semple et al., 2010; Hussmann et al., 2013). Among the CCR2 ligands, it appears that CCL2 and CCL7 play a dominant role in regulating CCR2-mediated monocytosis, while CCL12 was dispensable at the steady state (Tsou et al., 2007). It is unclear at the moment which of these chemokines (or both) is involved in WNV-induced monocytosis, and whether migration from the blood into the CNS requires either or both of these ligands.

Very little is known regarding the role of monocytes or CCR2 during other flavivirus or alphavirus infections. In the context of JEV and TBEV infection, CCL2 has been detected in the plasma and CSF of patients (Michałowska-Wender et al., 2006; Gupta et al., 2010); however, no studies have evaluated the role of monocytes or CCR2 in the context of these infections in mice. For alphavirus infections, we recently showed that the CCR2 ligand, CCL2, is highly inducible in the brain during SFV infection in mice (Michlmayr et al., 2014). The extent of CCL2 upregulation was correlated with the virulence of the strain, with the virulent strain of SFV inducing a >3000-fold induction above healthy control brains versus an ∼90-fold induction using the avirulent strain of SFV. Interestingly, the composition of the CNS infiltrate differed greatly between these two strains, with the avirulent strain inducing Ly6C^hi^ monocyte infiltration followed by a large influx of T-cells at the peak of infection. Conversely, the virulent strain of SFV induced an early and large influx of monocytes, with mice succumbing to infection by day 6, prior to when T-cells typically appear in the CNS. To investigate the role of CCR2 in this model, we infected *Ccr2*-deficient mice with virulent SFV but found no significant change in survival (data not shown). We also evaluated the therapeutic potential of CCR2 blockade during SFV infection using a CCR2-specific antagonist, RS504393, which is a highly selective small molecule inhibitor (Mirzadegan et al., 2000; Furuichi et al., 2003). Treatment of SFV-infected mice twice per day, starting on day 3, resulted in no significant change in survival compared to untreated mice (Michlmayr et al., 2014). In both *Ccr2^-/-^* mice and RS504393-treated mice, drastically reduced monocyte numbers were observed in the CNS compared to control-infected mice. Interestingly, brain viral titers in *Ccr2^-/-^* and RS504393-treated versus control mice were not significantly altered (**Table 1**), suggesting that monocytes may not contribute to viral clearance in the CNS (Michlmayr et al., 2014). Together, these results suggest that monocytes, although they migrate into the CNS during SFV infection of mice, may not impact pathogenesis. More work is required to fully understand monocyte migration and function during SFV and other alphavirus infections.

#### CCR2 receptor antagonists

There is great interest in developing a CCR2-specific antagonist due to pathogenic roles of monocytes in a wide range of inflammatory diseases (Bachelerie et al., 2013). Currently, several small molecule receptor antagonists have made it successfully into clinical trials, including JNJ-17166864 developed by Johnson and Johnson, CCX140 from ChemoCentryx, and a CCR2-neutralizing antibody from Millenium (Hou et al., 2008; Hanefeld et al., 2012; Bachelerie et al., 2013). Based on our current understanding of how monocytes function during viral infection of the CNS, it would be expected that blocking monocyte entry into the CNS via CCR2 blockade, either by preventing their egress from the bone marrow or their migration into the CNS, could be detrimental to the patient during natural infection. However, our studies using SFV infection as a model of neurotropic alphavirus infection suggest that CCR2 blockade in this context may have no effect (Michlmayr et al., 2014).

### CCR5

**FIGURE 2:**
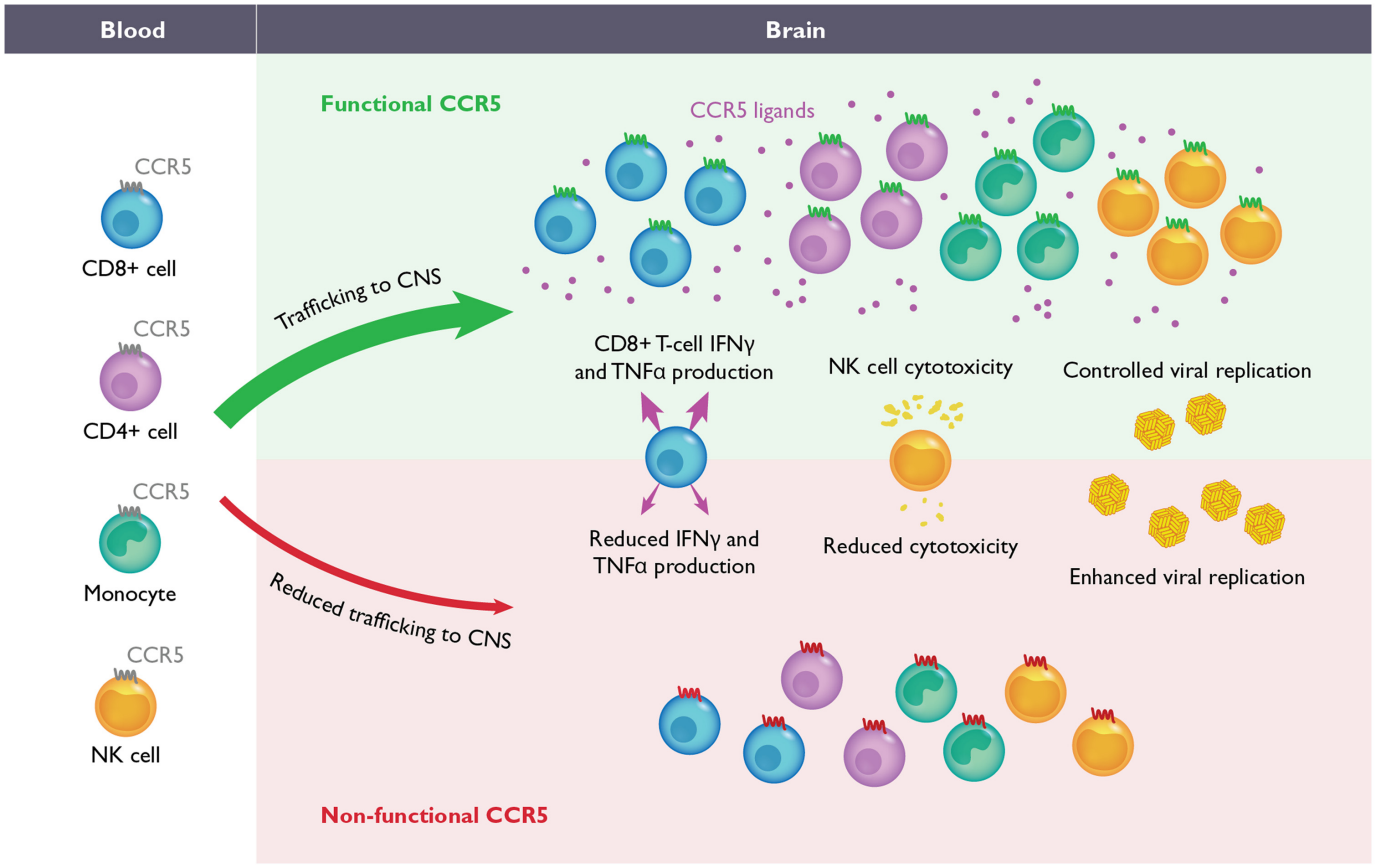
**The role of CCR5 during flavivirus-induced encephalitis**. During WNV and Japanese encephalitis virus (JEV) infection in mice, the migration of CD4^+^ T-cells, CD8^+^ T-cells, NK-cells and monocytes from the blood into CNS is required for efficient control of viral replication and recovery (upper). In the absence of CCR5 in mice, the migration of these leukocytes into the CNS is delayed and/or severely impaired (lower). During JEV infection, the absence of CCR5 also results in inefficient NK and CD8^+^ T-cell effector functions as well. Although the function of CCR5 has not been evaluated during WNV infection in humans, homozygosity for the complete loss-of-function mutation, *CCR5Δ32*, has been correlated with increased severity of WNV and tick borne encephalitis virus infections. It is anticipated that blockade of CCR5, either in mice or humans, may increase susceptibility to neurotropic flaviviruses.

Due to its role as an HIV-1 co-receptor, CCR5 is one of the most highly studied chemokine receptors to date (Deng et al., 1996; Dragic et al., 1996). This receptor is mainly expressed on subsets of activated T-cells, NK-cells and myeloid cells (**Figure 2**), including monocytes, DCs, and microglia (Mack et al., 2001). Until recently, the function of CCR5 in host defense in humans was thought to be redundant with other closely related receptors, since individuals genetically deficient for CCR5 showed no increased susceptibility to infectious agents (Lim et al., 2006). However, recent studies have provided evidence that CCR5 may have a neuroprotective role during acute flaviviral infections of the CNS in both mice and humans.

The CCR5 ligands, CCL3, CCL4, and CCL5 have been shown to be among the most highly upregulated chemokines in the CNS during WNV, TBEV, JEV, and SFV infection in mice and humans (Glass et al., 2005; Klein et al., 2005; Palus et al., 2013; Michlmayr et al., 2014). The source of these ligands *in vivo* is unclear; however, *in vitro* studies, using primary cell cultures, suggest that, at least for CCL5, the major source could be astrocytes and possibly microglia (Cheeran et al., 2005; Chen et al., 2011; Hussmann and Fredericksen, 2014). The extent of CCL5 production may be dependent on the pathogenic potential of the virus, with astrocytes producing significantly higher levels of CCL5 when encountering a more virulent strain of WNV (Hussmann and Fredericksen, 2014). In WNV-infected plasma samples from blood donors during the viremic phase of infection, none of the CCR5 ligands were elevated above control levels (Tobler et al., 2008). However, in the context of JEV infection, CCL5 levels detected in human CSF appeared to be positively correlated with leukocyte numbers in the CSF. Additionally, plasma levels of CCL5 were significantly elevated in fatal compared to non-fatal human cases (Winter et al., 2004).

Pathogenesis studies in mice have revealed a critical role for CCR5 during flavivirus infection. In the context of WNV infection, significant differences were observed only within the CNS, with *Ccr5^-/-^* mice exhibiting increased viral loads, concomitant with reduced accumulation of infiltrating CD4^+^ and CD8^+^ T-cells, NK cells and monocytes/macrophages (Glass et al., 2005). The loss of these cells correlated with 100% mortality in *Ccr5^-/-^* mice compared to wild type controls, where survival was documented to be ∼35%. Of note, viral clearance from the spleen in both *Ccr5^-/-^* and wild type mice was identical and underlines the importance of a CNS specific role of CCR5 in WNV-infected mice. Adoptive transfer of CCR5^+^ splenocytes into *Ccr5^-/-^* mice restored the survival rate similar to that in wild type infected mice. These data strongly suggest that WNV infection within the CNS triggers a CCR5-dependent influx of leukocytes into the CNS that is required for viral clearance and survival (**Figure 2**).

Similar results, showing a critical role for CCR5 in host defense, have been obtained in a mouse model of JEV infection (Larena et al., 2012). Larena et al. reported that *Ccr5^-/-^* mice infected with JEV exhibit increased mortality (64%) compared to wild type mice (28%), correlating with higher viral titers in the CNS. Peripheral control of virus and the induction of the humoral immune response were similar between wild type and *Ccr5^-/-^* mice. However, NK and CD8^+^ T-cell effector functions were blunted in the absence of CCR5 compared to wild type mice (**Figure 2**). Rather than a loss of leukocyte infiltration, as observed in WNV infection, a transient delay in the recruitment of NK cells, CD4^+^ and CD8^+^ T-cells, and monocytes into the infected CNS was observed. Together, these data suggest that the functions of CCR5 are critical for controlling both leukocyte trafficking as well as effector functions, which is required for efficient clearance of virus from the CNS.

During SFV infection in mice, evaluation of chemokine transcript numbers in the CNS revealed high induction of CCL3, CCL4, and CCL5 expression, suggesting a possible role for CCR5 in pathogenesis of SFV (Michlmayr et al., 2014). Although *Ccr5^-/-^* mice have not been tested, we evaluated the efficacy of a small molecule CCR5 receptor antagonist, named DAPTA, in a mouse model of SFV. DAPTA is a synthetic peptide comprising eight amino acids (185–192) of the gp120 V2 region of HIV-1 that binds competitively to the ligand-binding site of CCR5 (Polianova et al., 2005). Mice were infected with either the avirulent or virulent strain of SFV and then treated subcutaneously once daily with DAPTA, starting on day 3 post infection. The results reveal no significant difference in mortality during virulent SFV infection and no significant reduction of leukocytes entering the brain of treated mice compared to untreated mice (both virulent and avirulent strains; **Table 1**). However, the number of CCR5 expressing cells in the brains of treated mice was significantly reduced compared to untreated mice, confirming the efficacy of DAPTA in blocking CCR5. Our data suggest that other chemokine receptors may be involved in attracting leukocytes into the virally infected brain during SFV infection.

#### Epidemiological studies

Based on the evidence in mouse models suggesting a critical neuroprotective role for CCR5 during flavivirus infections, several epidemiologic studies have been conducted to evaluate the role of CCR5 in humans, specifically testing for the complete loss-of-function mutation *CCR5Δ32*. This phenotype is commonly found at 10% allele frequency in the US (Zimmerman et al., 1997; Berger et al., 1999). In the context of WNV infection, several initial cohort studies, using patient samples collected from the US epidemic, showed a significant increase in *CCR5Δ32* homozygosity among WNV infected individuals with symptomatic outcome compared to uninfected controls (Glass et al., 2006; Lim et al., 2008, 2010). Further, the frequency of the *CCR5Δ32* mutation among WNV infected individuals who remained asymptomatic was significantly depressed compared to controls, suggesting that all WNV infected *CCR5Δ32* homozygotes progress to symptomatic disease (Lim et al., 2010). Evaluation of *CCR5Δ32* heterozygotes showed no increased susceptibility, suggesting that partial functionality of CCR5 was sufficient to confer protection (Lim et al., 2010). However, these findings have recently been challenged by two additional studies, which failed to find any association of symptomatic WNV infection and *CCR5Δ32* mutation. Reasons for these discrepant results may be due to differences in study design, cohort size, and/or race composition of the tested cohorts and control populations (Bigham et al., 2011; Loeb et al., 2011). Further studies, using large and well characterized cohorts, in which symptom development is documented, should be conducted.

The frequency of the *CCR5Δ32* mutation has also been evaluated in a cohort of TBEV-infected patients (Kindberg et al., 2008). Genotyping for the *CCR5Δ32* mutation revealed an increased frequency among TBE patients, compared to the aseptic meningoencephalitis and control group. Furthermore, the allele frequency of *CCR5Δ32* correlated with disease severity. Although this study evaluated a small number of patients, these data suggest that the *CCR5Δ32* mutation may be a risk factor for developing severe disease after TBEV infection. In a follow up study by Barkhash et al. (2013) using a larger cohort, the authors found no significant association between *CCR5Δ32* mutation and predisposition to TBE.

Among the encephalitic flaviviruses, JEV is responsible for the most cases of encephalitis worldwide (Campbell et al., 2011). Although CCR5 is a critical host factor during JEV infection in mice, an evaluation of *CCR5Δ32* in patients would be difficult in cohorts of JEV-infected individuals, since endemic areas are expected to have a low or absent *CCR5Δ32* allele frequency (Zimmerman et al., 1997; Berger et al., 1999). However, there are several other CCR5 polymorphisms described in the literature that strongly modulate CCR5 expression, and genotype:phenotype association studies using these genetic probes are feasible (Carrington et al., 1999).

#### CCR5 receptor antagonists

Studies in mice, along with epidemiologic data, suggest that CCR5 is protective in WNV, JEV, and TBEV infection, which highlights the concern for chronic use of CCR5 antagonists in humans. Maraviroc is an FDA-approved CCR5 antagonist that is currently approved for the treatment of HIV-infected patients. Further, this antagonist is being considered for several other inflammatory diseases, with anticipated long-term use (Velasco-Velazquez et al., 2012; Wilkin and Gulick, 2012; Cipriani et al., 2013; Ochoa-Callejero et al., 2013). Based on epidemiologic data showing no change in susceptibility among *CCR5Δ32* heterozygotes, the risk of CCR5 blockade will depend on the amount of CCR5 coverage achieved by the drug. We would anticipate that cessation of CCR5 blockade should fully restore the functionality of this chemokine receptor, in the event that a patient prescribed CCR5 blockers develops symptoms associated with flavivirus infection. More studies will be required to fully understand the impact of CCR5 antagonists during flavivirus infection in humans.

### CXCR2

Neutrophils are an important cellular component of the innate immune response and appear to have a role in the pathogenesis of flavivirus infection of the CNS. During WNV encephalitis in mice, neutrophils comprise a significant proportion of the cellular infiltrate within the CNS (Lim et al., 2011). Likewise in humans, neutrophils were predominant in the CSF of patients with neuroinvasive disease (Rawal et al., 2006; Tyler et al., 2006). Like monocytes, neutrophils reside in the bone marrow, and their mobilization is regulated, in part by CXCR2, a chemokine receptor highly expressed on mouse and human neutrophils (Eash et al., 2010). In mice, CXCR2 binds CXCL1 and CXCL2, while in humans, this receptor binds most potently to CXCL8 (Murphy, 1997). A study by Bai et al. (2010) demonstrated that neutrophils play a dual role during WNV infection. Using a Gr-1 or Ly6G neutralizing antibody, the authors showed that depletion of neutrophils prior to infection resulted in reduced viral loads and enhanced survival compared to untreated mice. However, if neutrophils are depleted 1 or 2 days after infection, greater viremia and mortality was observed. These data suggest that, depending on the timing of depletion, neutrophils can be either beneficial and contribute to viral clearance or can be detrimental and increase viral dissemination of WNV in mice. The authors also evaluated the mortality rate between WNV-infected wild type and *Cxcr2^-/-^* mice, which revealed a significant increase in survival time in the absence of CXCR2 (Bai et al., 2010). Viremia was decreased on day 1 in *Cxcr2^-/-^* mice, compared to wild type mice; however, by day 3, viremia was higher in CXCR2 deficient mice. These data suggest that CXCR2 is involved in early migration steps that affect viral dissemination but may contribute to viral clearance during the later phases of infection. Unfortunately, the migration of neutrophils into the CNS was not measured in the absence of CXCR2, and more studies are required to understand the chemokine receptors required for their entry into the CNS and their function.

No mouse studies have addressed the role of neutrophils during JEV infection. However, several lines of evidence suggest that these cells are important *in vivo*. Firstly, JEV has been shown to induce neutrophilia in mice infected intraperitoneally; this has also been observed in human infections as well (Chaturvedi et al., 1979; Johnson et al., 1986; Mathur et al., 1988, 1992; Chung et al., 2007). Secondly, significant induction of CXCL8 levels was concomitant with the presence of neutrophils in the CSF of patients positive for JEV (Winter et al., 2004). Notably, significantly higher levels of CXCL8 were associated with patient mortality (Singh et al., 2000). Studies determining the signals involved in neutrophilia versus monocytosis, and how these are differentially regulated during JEV and WNV infection, could provide useful insights into the different clinical presentations observed in patients.

Murray Valley encephalitis virus (MVEV) is a mosquito-transmitted neurotropic flavivirus, endemic to parts of Australia and Papua New Guinea. Using a mouse model of MVEV infection, one study showed that the leukocytic infiltrate in the CNS predominantly comprised neutrophils, which appeared to colocalize with neurons and was preceded by the induction of CXCL1 expression (Andrews et al., 1999). Depletion of neutrophils, using a Gr-1 antibody, resulted in increased survival (55%) compared to isotype control-treated mice, where infection was uniformly lethal. These data suggest that the production of iNOS in the CNS may be the mechanism by which neutrophils are promoting pathogenesis in this model. No further studies were conducted to understand the temporal and organ-specific roles of neutrophils in promoting viral replication or to determine which receptor is utilized for tissue migration.

In the context of SFV infection, we showed that neutrophil-associated chemokines CXCL1 and CXCL2 were not expressed in the brain of mice infected with the avirulent strain. Consistent with this, no neutrophils were observed in the CNS as assessed by flow cytometric analysis and immunohistochemistry (Michlmayr et al., 2014). In contrast to this, CXCL2 transcripts were highly upregulated in murine brains infected with the virulent strain of SFV, exhibiting a >2000-fold increase in CXCL2 transcripts compared to healthy control brains. Neutrophils were detected in the CNS of virulent SFV-infected mice by histology, and the extent of CXCL2 upregulation correlated with disease severity, as lethally SFV-infected mice displayed higher CXCL2 induction in the CNS compared to asymptomatic mice (Michlmayr et al., 2014). The function of neutrophils in this model is unclear, and more studies are needed to understand their role, if any, in SFV and other neurotropic alphavirus infections.

#### CXCR2 receptor antagonists

Because of the dominant role of CXCL8 in the activation and recruitment of neutrophils in humans, several antagonists for its receptors CXCR1 and CXCR2 have been developed for a wide range of diseases, including COPD, cystic fibrosis, and pancreatic islet transplantation (Horuk, 2009; Bachelerie et al., 2013). Reparixin, a non-competitive allosteric inhibitor of CXCR1 and CXCR2, appears to be the farthest along, having now entered clinical phase III trials in Europe and the United States (Citro et al., 2012). Several others are also in the pipeline, including ones specific for CXCR2 (Dwyer et al., 2006; Chapman et al., 2007; Gonsiorek et al., 2007). The function of neutrophils during flavivirus infection, both in the periphery and CNS, is unclear. However, since depletion of neutrophils prior to infection promotes survival, individuals chronically administered CXCR1/2 antagonists may experience some level of protection during WNV infection. In the rare event that an individual is administered a CXCR1/2 blocker soon after infection, increased WNV replication in the periphery could result in a more aggressive disease outcome.

### CXCR3

**FIGURE 3:**
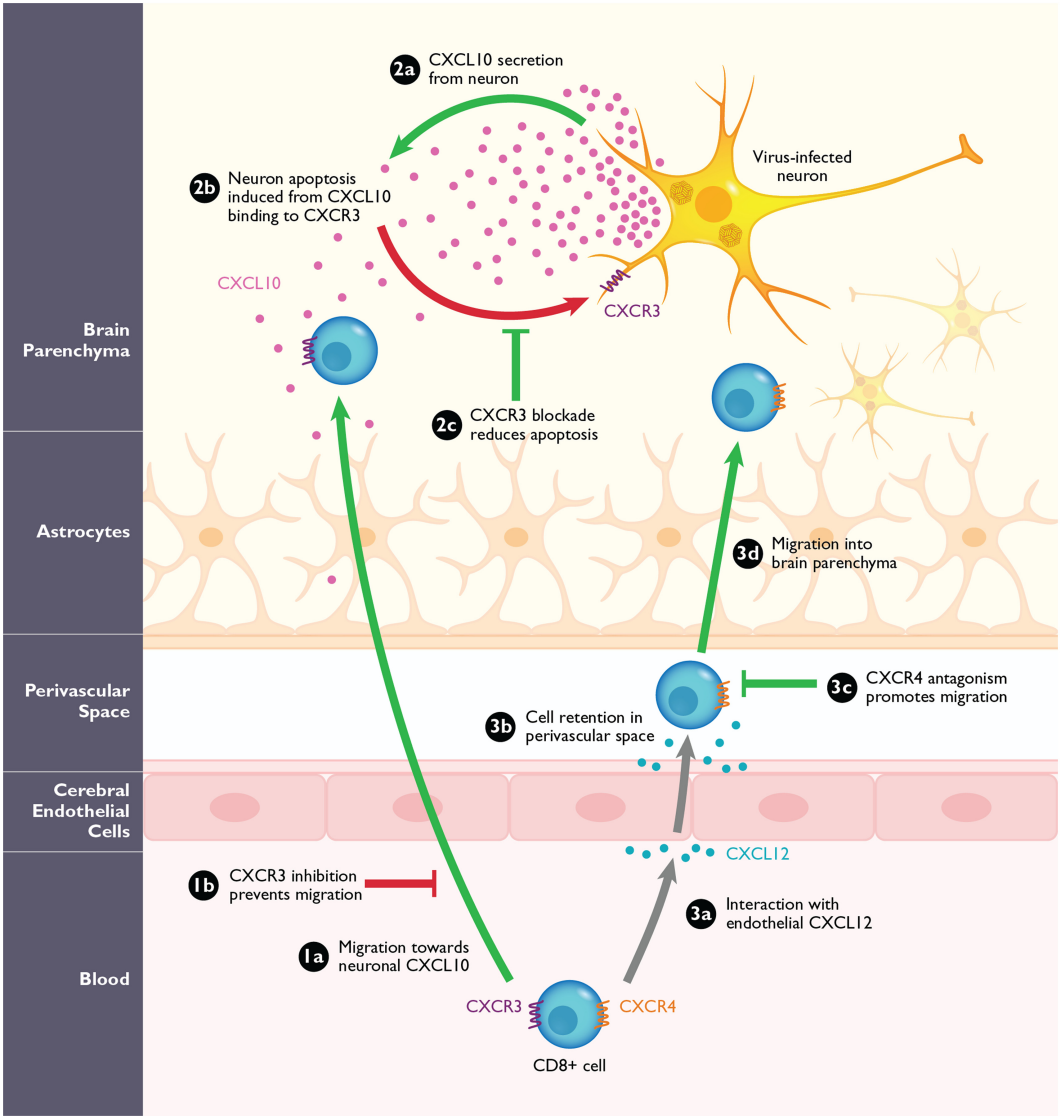
**The role of CXCR3 and CXCR4 during arbovirus-induced encephalitis in mice**. **(1a)** CXCR3^+^CD8^+^ T-cells migrate into the CNS in response to high levels of neuronal CXCL10. Loss of CXCR3 or its ligand CXCL10, or antagonizing this interaction **(1b)** prevents the migration of CD8^+^ T-cells into the CNS during WNV in mice. **(2a)** Virally infected neurons are the predominant source of CXCL10 in the CNS during WNV infection. **(2b)** Neuronal CXCL10 can engage CXCR3 that is upregulated on infected neurons and induce apoptosis. **(2c)** Blockade of CXCR3 with antagonists or CXCL10 neutralizing antibodies leads to the reduced binding of neuronal CXCL10 to neuronal CXCR3 and result in reduction of apoptosis and increased neuronal survival. **(3a)** During WNV infection, CXCR4^+^CD8^+^ T-cells migrate toward endothelial CXCL12, expressed on the inflamed cerebral endothelium. **(3b)** The interaction of CXCL12 and CXCR4 causes the CD8^+^ T-cells to be retained within the perivascular space. **(3c,d)** After blockade with the CXCR4 antagonist, AMD3100, the CD8^+^ T-cells are released and can migrate into the brain parenchyma where they promote clearance of WNV. Pathological steps are depicted in red; beneficial steps involved in increasing survival and improving disease outcome in the host are depicted in green. Gray arrows signify functions that are neither beneficial nor pathogenic.

The chemokine receptor CXCR3 is found at high levels on activated T-cells and NK-cells, and it can bind to chemokine ligands CXCL9, CXCL10, or CXCL11. The induction of these ligands is nearly always associated with cell-mediated immunity, and these chemokines are considered to be interferon-stimulated genes. The recruitment of antigen-specific T-cells is a critical step in viral clearance within the CNS, and the CXCL10:CXCR3 axis appears to be particularly important in this process, at least in the context of WNV (**Figure 3**). CXCL10 is the most highly induced chemokine in the CNS in mouse models of WNV, JEV, TBEV, and SFV encephalitis (Klein et al., 2004; Glass et al., 2005; Garcia-Tapia et al., 2007; Cao et al., 2011; Yang et al., 2011; Palus et al., 2013; Michlmayr et al., 2014). In addition, the other CXCR3 ligands, CXCL9 and CXCL11, are also often upregulated in the infected CNS, although no studies have evaluated the role of these chemokines *in vivo* (Klein et al., 2004; Glass et al., 2005; Michlmayr et al., 2014).

The most thorough analyses of CXCL10 and CXCR3 come from data collected for WNV infection. In WNV-infected blood donors, CXCL10 is significantly upregulated in the plasma during the viremic phase of infection, with levels significantly decreased after seroconversion, consistent with its role as an interferon-stimulated gene (Tobler et al., 2008). Although *in vitro* studies using primary brain cell cultures, showed CXCL10 expression in WNV-infected astrocytes and microglia, the production of CXCL10 *in vivo* appears to be primarily by infected neurons (Cheeran et al., 2003; Shirato et al., 2004; Klein et al., 2005). Using CXCL10-deficient mice and an anti-CXCL10 neutralizing antibody, Klein et al. (2005) observed a significant reduction in CD8^+^ T-cells within the CNS, higher viral titers in the brain, and enhanced mortality compared with wild type mice (**Table 1**). These results were phenocopied using *Cxcr3^-/-^* mice (Zhang et al., 2008). Of note, the number of CD4^+^ T-cells was not significantly reduced in the WNV-infected brain, suggesting that CXCL10 is important for the specific recruitment of CD8^+^ T-cells and that other receptors may compensate for the recruitment of CD4^+^ T-cells (**Figure 3**). Additionally, the effect of CXCL10 on the pathogenesis of WNV infection appears to be CNS-specific since clearance of WNV in peripheral tissues were identical compared to wild type infected mice (Klein et al., 2005). Thus, these data show an indispensable role for CXCL10 in the recruitment of CXCR3-expressing CD8^+^ T-cells into the CNS.

In addition to the role of the CXCL10:CXCR3 axis in regulating CD8^+^ T cell migration into the CNS, a more recent study has demonstrated a role for this chemokine:receptor pair in promoting neuronal apoptosis during WNV encephalitis. The authors found that TNF-α produced during WNV encephalitis caused specific downregulation of CXCR3 expression on infected and bystander neurons. Downregulation of CXCR3 through pretreatment with TNF-α or in *Cxcr3^-/-^* mice resulted in increased neuronal survival through delayed activation of caspase 3. These data suggest that although CXCL10:CXCR3 signaling is protective in its capacity to promote effector CD8^+^ T-cell migration to assist with viral clearance, the same signaling event on neurons may induce neuronal apoptosis and exacerbate immune-mediated damage (**Figure 3**). These data exemplify the complexity of chemokines during CNS inflammation and suggest that the use of CXCR3-specific antagonists may be beneficial or detrimental, depending on cell-type specific effects and timing.

In TBEV-infected patients presenting with neuroinvasive disease, two studies reported high levels of CXCL10 in the CSF (Lepej et al., 2007; Zajkowska et al., 2011); cytoanalysis of CSF samples revealed that the majority of CD4^+^ T-cells were positive for CXCR3. These data suggest that the CXCL10:CXCR3 axis may also be critical for T-cell migration into the CNS. Although CXCL10 expression is highly upregulated in the CNS during TBEV infection in mice, the specific role of these cells in directing T-cell migration into the CNS has not been studied (Tigabu et al., 2010; Palus et al., 2013).

In the context of alphavirus infection, the role of CXCR3 has not been evaluated yet. CXCL9 and CXCL10 are highly upregulated in the CNS of SINV-infected mice, and preceded the infiltration of T-cells and B-cells (Metcalf et al., 2013). Flow cytometric analyses of B-cells within the SINV infected CNS revealed high and uniform expression of CXCR3 on these cells. During SFV infection with both virulent and avirulent strains, CXCL9 and CXCL10 were highly upregulated in the CNS of infected mice as well, correlating with a large influx of T-cells, primarily CD8^+^ T-cells, into the CNS (Michlmayr et al., 2014). To evaluate the role of CXCR3 in the context of SFV infection, we employed a small molecule antagonist, compound 21, an imidazo-pyrazine derivative, which has been shown to block binding of CXCL9, CXCL10, and CXCL11, and is specific for CXCR3 (Du et al., 2009). SFV-infected mice were treated subcutaneously once daily starting on day 3 post infection. Treatment of avirulently infected mice with compound 21 resulted in a significant decrease of T-cell infiltration into the CNS compared to untreated mice, suggesting that CXCR3 is involved in the T-cell migration into the inflamed brain (**Figure 3**). Surprisingly, blockade of CXCR3 during lethal SFV infection did not result in a change in survival (**Table 1**). However, the combined blockade of CXCR3 and CCR2, did result in significantly enhanced survival compared to untreated mice (**Table 1**). Thus, the dual blockade of CXCR3 and CCR2 is necessary to achieve a survival benefit during lethal SFV-induced encephalitis. It is likely that these antagonists are inhibiting both T-cells and monocytes, implying that lethality in this model is immune-mediated. Furthermore, our data suggest that dual blockade of chemokine receptors may be more effective for treating diseases, a paradigm known as polypharmacology (Roth et al., 2004; Frantz, 2005; Overington et al., 2006). This concept is reviewed in more detail elsewhere (Overington et al., 2006; Horuk, 2009).

#### CXCR3 receptor antagonists

Compound 21, synthesized by Amgen, has been shown to bind to CXCR3 with high potency, inhibiting the binding of cognate CXCR3 ligands (Du et al., 2009). In addition to this compound, there are several other CXCR3 blockers in clinical development for the treatment of rheumatoid arthritis and psoriasis and is extensively reviewed elsewhere (Bachelerie et al., 2013; Michlmayr and McKimmie, 2014). At least during WNV infection, there appears to be a dual function of the CXCR3:CXCL10 axis; firstly, for the recruitment of antiviral CD8^+^ T-cells into the CNS, and secondly, to promote neuronal apoptosis (Klein et al., 2005; Bhowmick et al., 2007; Zhang et al., 2008). Despite this, the effect of global CXCR3 and CXCL10 deficiency resulted in increased mortality, suggesting that the dominant protective function of CXCR3 is at the level of CD8^+^ T-cell migration into the CNS, which strongly outweighs its role in promoting neuronal apoptosis (Zhang et al., 2008, 2010). Thus, we anticipate that the blockade of CXCR3 during flavivirus induced encephalitis may promote pathogenesis. In addition to CXCR3 antagonists, a neutralizing CXCL10 antibody, MDX-1100, is also being tested by Medarex for the treatment of rheumatoid arthritis and is currently in clinical phase II studies (Yellin et al., 2012). Similar to CXCR3 antagonism, MDX-1100 may block effector T-cell migration into the CNS and increase pathogenesis.

### CXCR4

CXCR4 and its sole ligand CXCL12 are among the most highly conserved in the chemokine superfamily (Lee et al., 1999; Zlotnik et al., 2006). CXCR4 has multiple critical functions, including embryonic development, homeostasis, and lymphoid organ retention as well as in serving as a coreceptor for HIV-1. As a result of its role in HIV entry, a small molecule inhibitor, AMD3100, was identified as a potent and selective antagonist for CXCR4. Under the name Plerixafor or Mozobil, AMD3100 is now FDA-approved and used to mobilize stem cells in non-Hodgkin lymphoma and multiple myeloma patients.

The role of CXCL12:CXCR4 during CNS inflammation in flavivirus and alphavirus infections is not well characterized. Because of the known role of the CXCL12:CXCR4 axis in cellular retention within the bone marrow, McCandless et al. (2008) hypothesized that the interaction of CXCL12 could be an important retention signal for cells migrating into the CNS. Indeed, CD8^+^ T-cells are restricted at the BBB through interactions with endothelial CXCL12 (**Figure 3**). Interrupting this interaction through the continuous administration of AMD3100 from the initial time of WNV infection in mice, resulted in the release of CD8^+^ T-cells in the perivascular space, allowing subsequent migration of these cells into the brain parenchyma, and is leading to enhanced viral clearance and survival (**Figure 3**; **Table 1**). Importantly, the authors showed the same pattern of perivascular retention of T-cells through CXCL12 and CXCR4 expression patterns in WNV-infected patients with neuroinvasive disease (McCandless et al., 2008). The authors also showed that glial cell activation was decreased in AMD3100-treated mice that can subsequently minimize pathological immune activation within the CNS. These data are even more impressive and relevant, since AMD3100 would be expected to function similarly in infected humans due to high conservation of CXCR4 between mice and humans.

#### CXCR4 receptor antagonists

Since AMD3100 is already FDA-approved (Mozobil, Plerixafor), the use of this antagonist for the treatment of WNV-infected individuals is possible. In fact, McCandless et al. (2008) have demonstrated that treatment of WNV-infected mice with AMD3100, starting on day 4 post infection, led to a prolonged survival, although no overall survival benefit was observed. These data are still very encouraging since increasing survival time, along with supportive care or in combination with other therapeutics as they become available, may provide important therapeutic options. In mice and humans, AMD3100 is capable of increasing overall numbers of leukocytes in the blood, which may also contribute to enhanced survival (Capoccia et al., 2006; McDermott et al., 2011).

## CONCLUSION

The emergence and spread of arboviral infections in the past few decades has highlighted the unpredictable nature of human outbreaks and emphasizes the need for novel treatment and prevention measures. The recent outbreak of WNV disease in the United States, and its continued emergence in several regions within Europe are just a few examples (Centers for Disease Control and Prevention [CDC], 2013; Sambri et al., 2013). Although there are vaccines available for the prevention of JEV and TBEV infection, no vaccines exist for human WNV infection, and no specific therapeutics are currently available for the treatment of neuroinvasive diseases caused by any arbovirus. Therefore, there is an urgent need for novel intervention strategies, either in the form of antivirals or immunomodulators that can block viral replication, boost protective immune responses, and minimize CNS injury. Furthermore, these therapeutics should be efficacious after the onset of symptoms, when the virus has entered the CNS and neuroinvasive symptoms have developed in patients.

Leukocyte migration is critically important during all phases of viral replication *in vivo*, and it is apparent that the chemokine network plays an integral role in the generation of an effective host immune response in the CNS. It is clear that both innate and adaptive immune responses are required for responding to and counteracting viral replication and spread, and since naturally acquired arboviral infections are initiated in the periphery, the timing and magnitude of both innate, as well as T- and B-cell responses, are critical for efficient viral control once the virus has accessed the CNS (Samuel and Diamond, 2005; Diamond and Gale, 2012). However, viral infections within the CNS require a response that is rapid and effective, but is not excessively exuberant as this could cause collateral damage to functionally critical and non-renewable cell types. A suboptimal or excessive immune response, or insufficient timing of immune responses, could alter the outcome of arboviral infections. Studies investigating chemokine-mediated leukocyte trafficking as well as other non-trafficking related functions during arboviral infections have provided great insight into our understanding of viral pathogenesis. Despite the great redundancy in the system, critical and non-overlapping functions for specific chemokines and receptors have been identified. Indeed, the migration of cells from the blood into the CNS, activation of effector cell function, mobilization of leukocytes, and retention of cells in the perivascular space are just a few examples. Thus, manipulating chemokine receptors therapeutically is a particularly attractive means by which to modulate outcome of infection. However, it is important to note that nearly all of the data so far have been evaluated using knock-out systems in mice; thus, our knowledge of the role of chemokine receptors on specific subsets of cells is incomplete primarily due to the lack of conditional knock out systems for most chemokine receptors. As reagents become available, it is imperative to further our understanding of how each receptor functions in a cell-type and organ-specific manner. Furthermore, chemokine receptor antagonists may be more relevant to understand the effect of therapeutics on disease outcome compared to knock-out model systems.

There are many areas of research that have remained relatively unexplored with regards to arboviral encephalitides: What are the key chemokine-mediated events that take place in local draining lymph node? What receptors coordinate optimal B- and T-cell activation within lymphoid tissues? How do neutrophils mobilize and migrate into the CNS? Still many questions remain, and there are many cell types that have not been studied in the context of arboviral encephalitis, most notably microglia. Several studies have shown that microglia are highly activated and proliferate during WNV encephalitis, and studies *in vitro* have found that these cells respond to virus through the TLR-3 pathway (Glass et al., 2005; Town et al., 2006; Getts et al., 2008). However, the function of microglia during WNV encephalitis *in vivo* is currently unknown. Since these cells exclusively express CX_3_CR1 in the healthy CNS, and its ligand CX_3_CL1 is constitutively expressed by neurons, this receptor:ligand interaction is likely to function during infection (Harrison et al., 1998; Cardona et al., 2006; Combadiere et al., 2007). In a healthy brain, the interaction between CX_3_CL1:CX_3_CR1 is hypothesized to suppress certain aspects of microglial activity (Zujovic et al., 2000; Cardona et al., 2006). Based on studies in numerous other models, loss of the signal, as in the case of CX_3_CR1-deficiency, results in microglia displaying an increased propensity for activation and function (Liu et al., 1998; Soriano et al., 2002; Sunnemark et al., 2005; Cardona et al., 2006; Fuhrmann et al., 2010). How the loss of CX_3_CR1 expression on microglia alters the outcome of WNV and other arboviral infections of the CNS will provide critical insights into their role in neuropathogenesis.

The use of chemokine receptor antagonists is an active area for drug development but is complicated by their pleiotropic functions that may have opposing effects. One such example is the dual role of CXCR3 during WNV pathogenesis. It has been shown that CXCR3 is critical for CD8^+^ T-cell migration into the CNS and promotes viral clearance and survival. However, it has also been shown that neuronally expressed CXCR3, and its interaction with CXCL10 on WNV-infected neurons, promotes neuronal apoptosis. Thus, CXCR3 antagonism would have both a protective and pathogenic effect on disease outcome; the diametric consequences of CXCR3 blockade could result in unanticipated effects. Additionally, due to the positive and negative pressures that contribute to the evolution of chemokine receptors over time, the role of any given receptor could be beneficial in one setting and detrimental in another. This complexity is best illustrated with CCR5, which functions in promoting infection in the context of HIV-1 infection but has the reciprocal effect during WNV infection (Lim et al., 2006). Thus, CCR5 antagonism, using FDA-approved Maraviroc for the treatment of HIV-infected individuals, carries the cost of promoting symptomatic WNV disease.

It is pivotal to understand the underlying immunological mechanism of encephalitis in order to develop effective treatment of acute viral encephalitis. New insights into the role of chemokines and their receptors in these contexts are also informative for studies of brain inflammation caused by multiple sclerosis, Alzheimer’s or Parkinson’s disease. The successful development of CCR5 and CXCR4 antagonists in humans demonstrates that chemokine receptors are feasible and effective targets that have the capacity to modulate disease. In fact, studies in mice and humans predict that these two antagonists would have opposite effects in human WNV disease, with Maraviroc promoting symptomatic disease and CXCR4 blockers promoting survival during infection. Because most chemokine receptor antagonists currently being developed will likely be administered chronically, it is critical to understand how these therapies may affect the individual in the context of their specific infection. This is an important goal that has been and should continue to be tested in the laboratory setting. Moreover, it is important to note that data obtained in inbred mouse models may not be applicable to humans. Thus, treatment of infected individuals with chemokine receptor antagonists may not function as anticipated based on mouse studies and should be approached with caution. Despite the success of Maraviroc and Mozobil/Plerixafor, there have been many receptor antagonists that have failed in clinical trials. Due to the redundancy of the chemokine system, antagonists that inhibit more than one receptor or the use of several compounds in conjunction may prove beneficial.

## Conflict of Interest Statement

The authors declare that the research was conducted in the absence of any commercial or financial relationships that could be construed as a potential conflict of interest.

## ABBREVIATIONS

BBB: blood–brain barrier
BCSFB: blood–cerebrospinal fluid barrier
CHIKV: Chikungunya virus
CNS: central nervous system
CSF: cerebrospinal fluid
DC: dendritic cell
EEEV: Eastern equine encephalitis virus
FDA: Food and drug administration
HIV: human immunodeficiency virus
iNOS: inducible nitric oxide synthetase
JEV: Japanese encephalitis virus
MHC: major histocompatibility complex
MVEV: Murray valley encephalitis virus
SCID: severe combined immunodeficiency
SFV: Semliki Forest virus
SINV: Sindbis virus
TBEV: tick borne encephalitis virus
TLR: toll-like receptor
TNF-α: tumor necrosis factor alpha
VEEV: Venezuelan equine encephalitis virus
WEEV: Western equine encephalitis virus
WNV: West Nile virus
